# Controllable symmetry breaking solutions for a nonlocal Boussinesq system

**DOI:** 10.1038/s41598-019-56093-8

**Published:** 2019-12-23

**Authors:** Jinxi Fei, Zhengyi Ma, Weiping Cao

**Affiliations:** 1 Institute of Optoelectronic Technology, Lishui University, Lishui, 323000 China; 2 Department of Mathematics, Lishui University, Lishui, 323000 China

**Keywords:** Applied mathematics, Nonlinear phenomena

## Abstract

The generalized Boussinesq equation is a useful model to describe the water wave. In this paper, with the coupled Alice-Bob (AB) systems, the nonlocal Boussinesq system can be obtained via the parity and time reversal symmetry reduction. By introducing an extended Bäcklund transformation, the symmetry breaking rogue wave, symmetry breaking soliton and symmetry breaking breather solutions for a nonlocal Boussinesq system are obtained through the derived **Hirota bilinear form**. The residual symmetry and finite symmetry transformation of the nonlocal AB-Boussinesq system are also studied.

## Introduction

Recently, Lou proposed various intrinsic models named the Alice-Bob systems (ABSs) to describe two-place physical problems^1,2^. The technical assumption was so-called $\hat{P}$-$\hat{T}$-$\hat{C}$ principle with $\hat{P}$ (the parity), $\hat{T}$ (time reversal) and $\hat{C}$ (charge conjugation). These ABSs mainly involved the following integrable systems, such as the Korteweg-de Vries (KdV), the nonlinear Schrödinger (NLS) and the Ablowitz-Kaup-Newell-Segur (AKNS), the modified KdV (MKdV), the sine-Gordon (sG), the Kadomtsev-Petviashvili (KP) and the Toda lattice systems. As a result, the $\hat{P}$-$\hat{T}$-$\hat{C}$ invariant multiple soliton solutions were explicitly presented for the above ABSs. In fact, various physically important models possess $\hat{P}$-$\hat{T}$-$\hat{C}$ invariance and these symmetries are important in many mathematical and physical fields. Up to now, researchers at least have studied the following works through the $\hat{P}$-$\hat{T}$-$\hat{C}$ principle: for a nonlocal AB-KdV system, its exact solutions including ${P}_{s}{T}_{d}$ invariant and ${P}_{s}{T}_{d}$ symmetric breaking solutions were given through different methods^3^. These solutions possess rich structures, such as single soliton, infinitely many singular soliton, cnoidal wave and the interaction of the different coherent structures. Nonlocal integrable peakon equations were also constructed and shown to have peakon solutions through this principle^4^. A new elegant form of the N-soliton solutions of the AB-KdV system which derived from the nonlinear inviscid dissipative and barotropic vorticity equation in a *β*-plane channel was obtained^5^. This system was applied to the two correlated **monopole** blocking events which was responsible for the snow disaster in the winter of 2007/2008 happened in Southern China. On the basis of the Lie point symmetry, the nonlocal symmetry of the AB-mKdV system was gained and the explicit solution of the system was presented^6^. By using the Darboux transformation, some types of shifted parity and time reversal symmetry breaking solutions, including such localized structures as one-soliton, two-soliton, and rogue wave solutions were explicitly depicted for the AB-mKdV system^7^. This methodology also induced a large number of nonlinear systems, including some integrable ones, such as the NLS, (potential) KdV, (potential) KP and sG systems from nothing (trivial $0=0$ equation) by introducing the new idea: consistent correlated bang and other suitable “Dao” which were implied by some symmetric or antisymmetric operators^8^. From a two-vortex interaction model established from the nonlinear inviscid dissipative and barotropic vorticity equation in a *β*-plane channel, a general nonlocal variable coefficient KdV (VCKdV) equation with shifted parity and delayed time reversal was derived through the multi-scale expansion method^9^. A special approximate analytical solution of the original two-vortex interaction model was given to describe two correlated dipole blocking events with a lifetime. Exact solutions of a new general nonlocal modified KdV equation were obtained including periodic waves, kink waves, solitary waves, kink- and/or anti-kink-cnoidal periodic wave interaction solutions, which can be utilized to describe various two-place and time-delayed correlated events^10^. The two-place nonlocal integrable models were systematically extended to multi-place nonlocal integrable (and nonintegrable) nonlinear models by means of the discrete symmetry reductions of the coupled local systems^11^. Especially, various two-place and four-place nonlocal NLS systems and KP equations were obtained. An integrable two-place nonlocal real system with three different nonlocal properties was derived which one nonlocality was related to a special nonlocal KdV system, while the other two were associated with the nonlocal Boussinesq systems^12^. The multisoliton solutions for the nonlocal KdV and Boussinesq systems were investigated and the possible prohibitions on multisoliton solutions were also discussed.

Now, we focus our attention on the following generalized Boussinesq equation

1 $${u}_{tt}+\alpha {u}_{xx}+\beta {({u}^{2})}_{xx}+\gamma {u}_{xxxx}=0,$$

which was first introduced by Boussinesq in 1871 to describe the propagation of long waves in shallow water^13^. This Boussinesq Eq. (1) is a soliton equation solvable by inverse scattering which arises in several other physical applications including one-dimensional nonlinear lattice-waves, vibrations in a nonlinear string and ion sound waves in a plasma^14–17^. Also, the soliton-cnoidal wave interaction solutions of the Boussinesq Eq. (1) was described when $\alpha =\gamma =-\,1,\beta =-\,3$^18^. The above Boussinesq equation with minus fourth-order term had multiple soliton solutions, whereas the model with the plus fourth-order term had multiple complex soliton solutions when the second-order derivative ${u}_{xx}$ was deleted and $\beta =\pm \,1$^19^. Rational solutions of this equation and applications to rogue waves was shown when $\alpha =-\,\beta =1,\gamma =-\,\frac{1}{3}$^20^. The bilinear transformation method was proposed to find the rogue wave solutions for the above generalized Eq. (1) ^21^. Two exponential-type integrators were proposed and analyzed for the “good” Boussinesq equation, i.e. $\alpha =\beta =-\,1,\gamma =1$ with rough initial data^22^. The Lie symmetry analysis was applied and some special solitons such as dark, singular and periodic solitons was devoted^23^.

The next several sections, we focus on the AB-Boussinesq system of the generalized Eq. (1). In Section 2, a nonlocal AB-Boussinesq system is constructed and its **bilinear form** is written through an extended Bäcklund transformation. In Section 3, the symmetry breaking rogue wave, symmetry breaking soliton and symmetry breaking breather solutions are presented through the derived **Hirota bilinear form**. Starting from a modified Bäcklund transformation, the residual symmetry of the system is studied by introducing two auxiliary variables in Section 4. Therefore, the finite symmetry transformation is obtained through solving the initial value problem. Some conclusions are given in the final section.

## Methods

### A nonlocal AB-Boussinesq system and its bilinear form

In this section, the model of AB system is applied into the Boussinesq equation. Based on the principle of the AB system referred in^1,2^, when substituting $u=\frac{1}{2}(A+B)$ into (1), the nonlocal AB-Boussinesq system is

2 $${A}_{tt}+{B}_{tt}+\alpha ({A}_{xx}+{B}_{xx})+\beta {({A}_{x}+{B}_{x})}^{2}+\,\beta (A+B)({A}_{xx}+{B}_{xx})+\gamma ({A}_{xxxx}+{B}_{xxxx})=0,$$

which can be split into two equatuions

3a $${A}_{tt}+\alpha {A}_{xx}+\beta (A+B){A}_{xx}+\frac{1}{2}\beta {({A}_{x}+{B}_{x})}^{2}+\gamma {A}_{xxxx}+G(A,B)=0,$$

3b $${B}_{tt}+\alpha {B}_{xx}+\beta (A+B){B}_{xx}+\frac{1}{2}\beta {({A}_{x}+{B}_{x})}^{2}+\gamma {B}_{xxxx}-G(A,B)=0,$$

where *B* is related to *A* by $B={\hat{P}}_{s}{\hat{T}}_{d}A=A(\,-\,x+{x}_{0},-\,t+{t}_{0})$ (${\hat{P}}_{s}{\hat{T}}_{d}$ expresses parity with a shift and time reversal with a delay). $G(A,B)$ is an arbitrary function of *A* and *B*, but should be ${\hat{P}}_{s}{\hat{T}}_{d}$ invariant. Although there are infinite formulas satisfying this $G(A,B)$ in Eq. (3), we take $G(A,B)=0$ for simplicity, and Eq. (3) are reduced to the following AB-Boussinesq system

4a $${A}_{tt}+\alpha {A}_{xx}+\beta (A+B){A}_{xx}+\frac{1}{2}\beta {({A}_{x}+{B}_{x})}^{2}+\gamma {A}_{xxxx}=0,$$

4b $${B}_{tt}+\alpha {B}_{xx}+\beta (A+B){B}_{xx}+\frac{1}{2}\beta {({A}_{x}+{B}_{x})}^{2}+\gamma {B}_{xxxx}=0.$$

Now, we introduce an extended Bäcklund transformation

5 $$A=\frac{6\gamma }{\beta }{(\mathrm{ln}f)}_{xx}+b{(\mathrm{ln}f)}_{x},\,B=\frac{6\gamma }{\beta }{(\mathrm{ln}f)}_{xx}-b{(\mathrm{ln}f)}_{x},$$

with *b* being an arbitrary constant and $f\equiv f(x,t)$ is an undetermined function of *x* and *t*. When $b=0$, Eq. (5) becomes one normal Bäcklund transformation. Substituting Eq. (5) into Eq. (4), the bilinear form can be derived as follows

6 $$({D}_{t}^{2}+\alpha {D}_{x}^{2}+\gamma {D}_{x}^{4})(f\cdot f)=0,$$

where ${D}_{t}^{2}$, ${D}_{x}^{2}$ and ${D}_{x}^{4}$ are the bilinear derivative operators defined by^24,25^

7 $${D}_{x}^{m}{D}_{t}^{n}(f\cdot g)={(\frac{\partial }{\partial x}-\frac{\partial }{\partial x^{\prime} })}^{m}{(\frac{\partial }{\partial t}-\frac{\partial }{\partial t^{\prime} })}^{n}\times f(x,t)g(x^{\prime} ,t^{\prime} ){|}_{x^{\prime} =x,t^{\prime} =t}.$$

According to the properties of bilinear operator *D*, Eq. (6) is equal to

8 $$f{f}_{tt}-{f}_{t}^{2}+\alpha (f{f}_{xx}-{f}_{x}^{2})+\gamma (f{f}_{xxxx}-4{f}_{x}{f}_{xxx}+3{f}_{xx}^{2})=0,$$

which happens to be a bilinear form of Eq. (1).

## Symmetry breaking rogue wave, soliton and breather solutions of the AB-Boussinesq system

In this section, we turn our attention to the **Hirota bilinear form** (6) of the nonlocal AB-Boussinesq system (4) to derive the symmetry breaking rogue wave, symmetry breaking soliton and symmetry breaking breather solutions.

### Symmetry breaking rogue wave solutions

In nonlinear science, especially for some nonlinear integrable systems which as one of the essential topics, the theoretical study of rogue waves has gotten more and more attention in recent years. Rogue waves, also known as freak waves, monster waves, extreme waves, or hundred year waves, are relatively large and spontaneous ocean surface waves, which are a serious threat even to large ships and ocean liners^26,27^. These phenomena are ubiquitous in nature and can appear in a variety of different contexts such as reported in liquid Helium, nonlinear optics and microwave cavities^28^.

For describing the symmetry breaking rogue wave solutions, we seek solutions of Eq. (4) in the form

9 $${A}_{n}=\frac{6\gamma }{\beta }{(\mathrm{ln}{f}_{n})}_{xx}+b{(\mathrm{ln}{f}_{n})}_{x},\,{B}_{n}=\frac{6\gamma }{\beta }{(\mathrm{ln}{f}_{n})}_{xx}-b{(\mathrm{ln}{f}_{n})}_{x},\,n\ge 1,$$

where ${f}_{n}\equiv {f}_{n}(x,t)$ is a polynomial of degree $\frac{1}{2}n(n+1)$ in *X*^2^ and *T*^2^ described by

10 $${f}_{n}=\mathop{\sum }\limits_{m=0}^{n(n+1)/2}\,\mathop{\sum }\limits_{j=0}^{m}\,{a}_{j,m}{(x-\frac{{x}_{0}}{2})}^{2j}{(t-\frac{{t}_{0}}{2})}^{2(m-j)}$$

with total degree $\frac{1}{2}n(n+1)$ and the constants ${a}_{j,m}$ $(j=0,1,\ldots ,m,m=0,1,\ldots ,\frac{n(n+1)}{2})$ are determined by equating powers of *X* and *T*^20^. Utilizing the **Hirota bilinear form** (6), we derive the following polynomials

11a $${f}_{1}=1-\frac{\alpha }{3\gamma }{X}^{2}-\frac{{\alpha }^{2}}{3\gamma }{T}^{2},$$

11b $${f}_{2}=1-\frac{{\alpha }^{3}{X}^{6}}{1875{\gamma }^{3}}+\frac{{\alpha }^{2}{\Delta }_{1}{X}^{4}}{1875{\gamma }^{3}}+\frac{\alpha {\Delta }_{2}{X}^{2}}{1875{\gamma }^{3}}-\frac{{\alpha }^{6}{T}^{6}}{1875{\gamma }^{3}}+\frac{17{\alpha }^{4}{T}^{4}}{1875{\gamma }^{2}}-\frac{19{\alpha }^{2}{T}^{2}}{75\gamma },$$

11c $$\begin{array}{rcl}{f}_{3} & = & 1+\frac{9{\alpha }^{6}{X}^{12}}{878826025{\gamma }^{6}}-\frac{18{\alpha }^{5}{\Delta }_{3}{X}^{10}}{878826025{\gamma }^{6}}+\frac{27{\alpha }^{4}{\Delta }_{4}{X}^{8}}{175765205{\gamma }^{6}}\\  &  & -\,\frac{12{\alpha }^{3}{\Delta }_{5}{X}^{6}}{175765205{\gamma }^{6}}-\frac{3{\alpha }^{2}{\Delta }_{6}{X}^{4}}{175765205{\gamma }^{6}}-\frac{6\alpha {\Delta }_{7}{X}^{2}}{878826025{\gamma }^{6}}\\  &  & +\,\frac{9{\alpha }^{12}{T}^{12}}{878826025{\gamma }^{6}}-\frac{522{\alpha }^{10}{T}^{10}}{878826025{\gamma }^{5}}+\frac{7803{\alpha }^{8}{T}^{8}}{175765205{\gamma }^{4}}\\  &  & -\,\frac{68484{\alpha }^{6}{T}^{6}}{25109315{\gamma }^{3}}+\frac{40143{\alpha }^{4}{T}^{4}}{717409{\gamma }^{2}}-\frac{870{\alpha }^{2}{T}^{2}}{847\gamma },\end{array}$$

where

12a $$\begin{array}{l}{\Delta }_{1}=25\gamma -3{\alpha }^{2}{T}^{2},\\ {\Delta }_{2}=125{\gamma }^{2}+90{\alpha }^{2}\gamma {T}^{2}-3{\alpha }^{2}{T}^{4},\\ {\Delta }_{3}=49\gamma -3{\alpha }^{2}{T}^{2},\\ {\Delta }_{4}=49{\gamma }^{2}-46{\alpha }^{2}\gamma {T}^{2},\end{array}$$

12b $$\begin{array}{rcl}{\Delta }_{5} & = & 3773{\gamma }^{3}-2793{\alpha }^{2}{\gamma }^{2}{T}^{2}+231{\alpha }^{4}\gamma {T}^{4}-3{\alpha }^{6}{T}^{6},\\ {\Delta }_{6} & = & 1037575{\gamma }^{4}+132300{\alpha }^{2}{\gamma }^{3}{T}^{2}-22470{\alpha }^{4}{\gamma }^{2}{T}^{4}\\  &  & +\,876{\alpha }^{6}\gamma {T}^{6}-9{\alpha }^{8}{T}^{8},\end{array}$$

12c $$\begin{array}{rcl}{\Delta }_{7} & = & 79893275{\gamma }^{5}-848925{\alpha }^{2}{\gamma }^{4}{T}^{2}\\  &  & -\,22050{\alpha }^{4}{\gamma }^{3}{T}^{4}-53130{\alpha }^{6}{\gamma }^{2}{T}^{6}\\  &  & +\,855{\alpha }^{8}\gamma {T}^{8}-9{\alpha }^{10}{T}^{10},\end{array}$$

with

13 $$X=x-\frac{{x}_{0}}{2},\,T=t-\frac{{t}_{0}}{2}.$$

**Figure 1:**
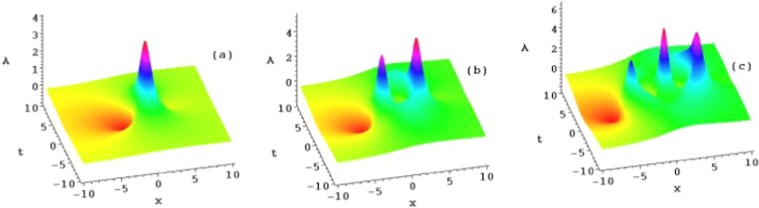
Plots of the symmetry breaking rogue wave solutions *A*_*n*_, for $n=1,2,3$, of the AB-Boussinesq system.

**Figure 2:**
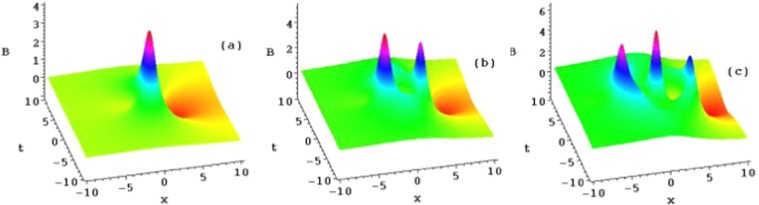
Plots of the symmetry breaking rogue wave solutions *B*_*n*_, for $n=1,2,3$, of the AB-Boussinesq system.

**Figure 3:**
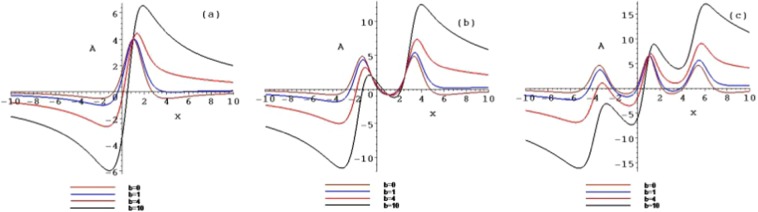
The contour plots of the symmetry breaking rogue wave solutions *A*_*n*_, for $n=1,2,3$, with $b=0,b=1,b=4$ and $b=10$ at the time $t=1$.

**Figure 4:**
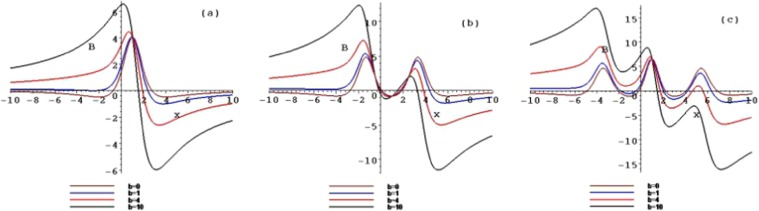
The contour plots of the symmetry breaking rogue wave solutions *B*_*n*_, for $n=1,2,3$, with $b=0,b=1,b=4$ and $b=10$ at the time $t=1$.

The following four sets of figures (Figs. 1, 2, 3 and 4) are presented for the purposes of illustration the solutions *u*_1_, *u*_2_ and *u*_3_. Figure 1 shows three ranks of the symmetry breaking rogue wave solutions ${A}_{n}(n=1,2,3)$ in Eq. (9) of the AB-Boussinesq system when the constants are $b=1,\alpha =1,\beta =\gamma =-\,1$ and ${x}_{0}={t}_{0}=2$^1–3,29^. Figure 2 depicts the reversal structures of Fig. 1 by another sets of solutions ${B}_{n}(n=1,2,3)$ (which are ${\hat{P}}_{s}{\hat{T}}_{d}$ symmetries of the solutions ${A}_{n}(n=1,2,3)$ in Eq. (9). These structures all possess two types of the rogue waves, namely, the bright and dark ones at the same time. This corresponds to the phenomenon that the shifted parity and delayed time reversal are applied to describe two-place events. We should mention that all the anti-symmetric solutions of Eq. (9) lead to the ${\hat{P}}_{s}{\hat{T}}_{d}$ symmetry breaking solutions of Eq. (4).

When selecting different parameter values *b*, the different structures of the above rogue waves can be described. Figures 3 and 4 show the contour plots of the symmetry breaking rogue waves in the $(x,A/B)$-plane, taking $b=0,b=1,b=4$ and $b=10$ at the time $t=1$, respectively. This parameter *b* can impact the wave shape. With the increase of *b* from 0 to 10, the amplitude is amplified, while the distance between the peaks is elongated. The parameter *b* plays an important role of stretching the rogue waves along with the *x* axis.

### Symmetry breaking soliton solutions

Based on the bilinear form (6) of the AB-Boussinesq system, the symmetry breaking multisoliton solutions of Eq. (4) can be written as a summation of some special functions^1,2,12^. For example, after choosing $\alpha =0,\beta =\gamma =-\,1$, we can take

14 $$\begin{array}{rcl}{f}_{n} & = & \sum _{\{\nu \}}\,{K}_{\{\nu \}}\,\cosh (\mathop{\sum }\limits_{i=1}^{n}\,{\nu }_{i}{\xi }_{i}),\\ {\xi }_{i} & = & {k}_{i}(x-\frac{{x}_{0}}{2})+2{\delta }_{i}{k}_{i}^{2}(t-\frac{{t}_{0}}{2}),\\ {\nu }_{i}^{2} & = & {\delta }_{i}^{2}=1,\end{array}$$

where the summation of $\{\nu \}=\{{\nu }_{1},{\nu }_{2},\ldots ,{\nu }_{n}\}$, and ${k}_{i}(i=1,2,\ldots ,n),{x}_{0},{t}_{0}$ are arbitrary constants, while

15 $${K}_{\{\nu \}}=\mathop{\prod }\limits_{i < j}^{n}\,{a}_{ij},\,{a}_{ij}^{2}=2{k}_{i}^{2}+2{k}_{j}^{2}-{k}_{i}{k}_{j}({\delta }_{i}{\delta }_{j}+3{\nu }_{i}{\nu }_{j}).$$

#### **Example 3**.**1**

Symmetry breaking line soliton solution

We take

16 $${f}_{1}=\,\cosh ({\xi }_{1}),\,{\xi }_{1}={k}_{1}(x-\frac{{x}_{0}}{2})+2{\delta }_{1}{k}_{1}^{2}(t-\frac{{t}_{0}}{2}),$$

with $n=1$. By substituting Eq. (16) into Eq. (5), one can get the symmetry breaking line soliton solution of Eq. (4)

17a $${A}_{1}=6{k}_{1}^{2}+b{k}_{1}\,\tanh ({\xi }_{1})-6{k}_{1}^{2}\,{\tanh }^{2}({\xi }_{1}),$$

17b $${B}_{1}=6{k}_{1}^{2}-b{k}_{1}\,\tanh ({\xi }_{1})-6{k}_{1}^{2}\,{\tanh }^{2}({\xi }_{1}).$$

Because of the existence of tanh term which makes ${B}_{1}={\hat{P}}_{s}{\hat{T}}_{d}{A}_{1}$ or ${A}_{1}={\hat{P}}_{s}{\hat{T}}_{d}{B}_{1}$, the line soliton solution (17) of the AB-Boussinesq system (4) is ${\hat{P}}_{s}{\hat{T}}_{d}$ symmetric breaking solution.

**Figure 5:**
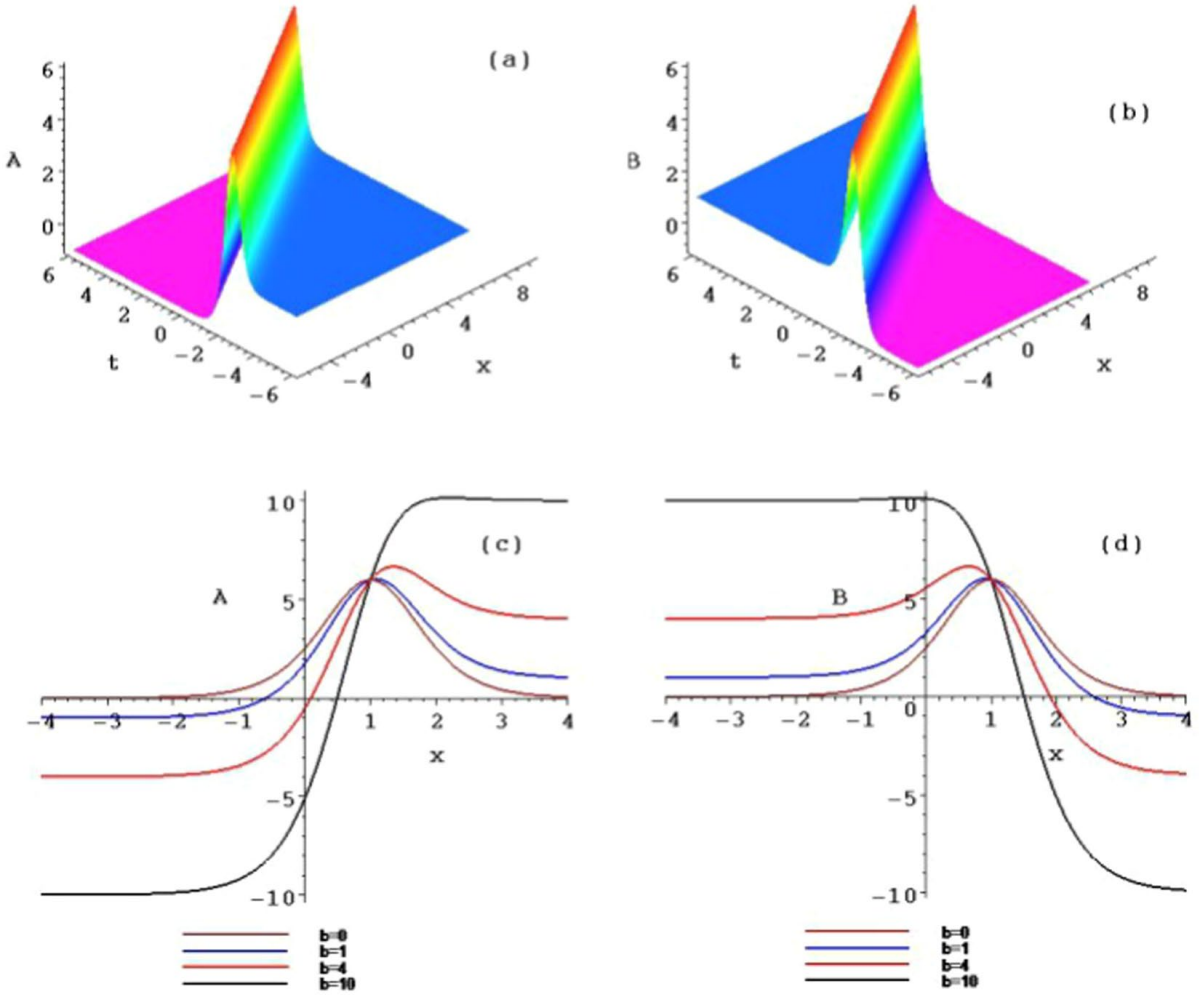
Plots of the symmetry breaking line solitons of *A*_1_ and *B*_1_ of the solution (17).

Figure 5(a,b) are two corresponding symmetric breaking structures when the constants taken as $b={k}_{1}=1,{\delta }_{1}=-\,1,{x}_{0}={t}_{0}=2$. Figure 5(c,d) are the contour plots of these structures in the $(x,A/B)$-plane, taking $b=0,b=1,b=4$ and $b=10$ at the time $t=1$, respectively. As the parameter *b* increases, the line soliton pairs trend to two symmetry breaking kinks.

#### **Example 3**.**2**

Interaction of the symmetry breaking line solitons

The interaction of the symmetry breaking line soliton solution of Eq. (4) can be expressed as

18 $${A}_{2}=6{(\mathrm{ln}{f}_{2})}_{xx}+b{(\mathrm{ln}{f}_{2})}_{x},\,{B}_{2}={\hat{P}}_{s}{\hat{T}}_{d}{A}_{2},$$

with

19 $$\begin{array}{rcl}{f}_{2} & = & {\delta }_{+}\sqrt{2{k}_{1}^{2}+2{k}_{2}^{2}-{k}_{1}{k}_{2}({\delta }_{1}{\delta }_{2}+3)}\,\cosh ({\xi }_{1}+{\xi }_{2})\\  &  & +\,{\delta }_{-}\sqrt{2{k}_{1}^{2}+2{k}_{2}^{2}-{k}_{1}{k}_{2}({\delta }_{1}{\delta }_{2}-3)}\,\cosh ({\xi }_{1}-{\xi }_{2}),\\ {\delta }_{+}^{2} & = & {\delta }_{-}^{2}=1,\end{array}$$

and

$${\xi }_{i}={k}_{i}(x-\frac{{x}_{0}}{2})+2{\delta }_{i}{k}_{i}^{2}(t-\frac{{t}_{0}}{2}),\,i=1,2.$$

When ${k}_{2}={k}_{1},{\delta }_{2}=-\,{\delta }_{1}$, Eq. (19) is simplified in the form

20 $$\begin{array}{rcl}{f}_{2} & = & {\delta }_{+}\sqrt{4{k}_{1}^{2}+{k}_{1}^{2}({\delta }_{1}^{2}-3)}\,\cosh [2{k}_{1}(x-\frac{{x}_{0}}{2})]\\  &  & +\,{\delta }_{-}\sqrt{4{k}_{1}^{2}+{k}_{1}^{2}({\delta }_{1}^{2}+3)}\,\cosh [4{\delta }_{1}{k}_{1}^{2}(t-\frac{{t}_{0}}{2})],\end{array}$$

**Figure 6:**
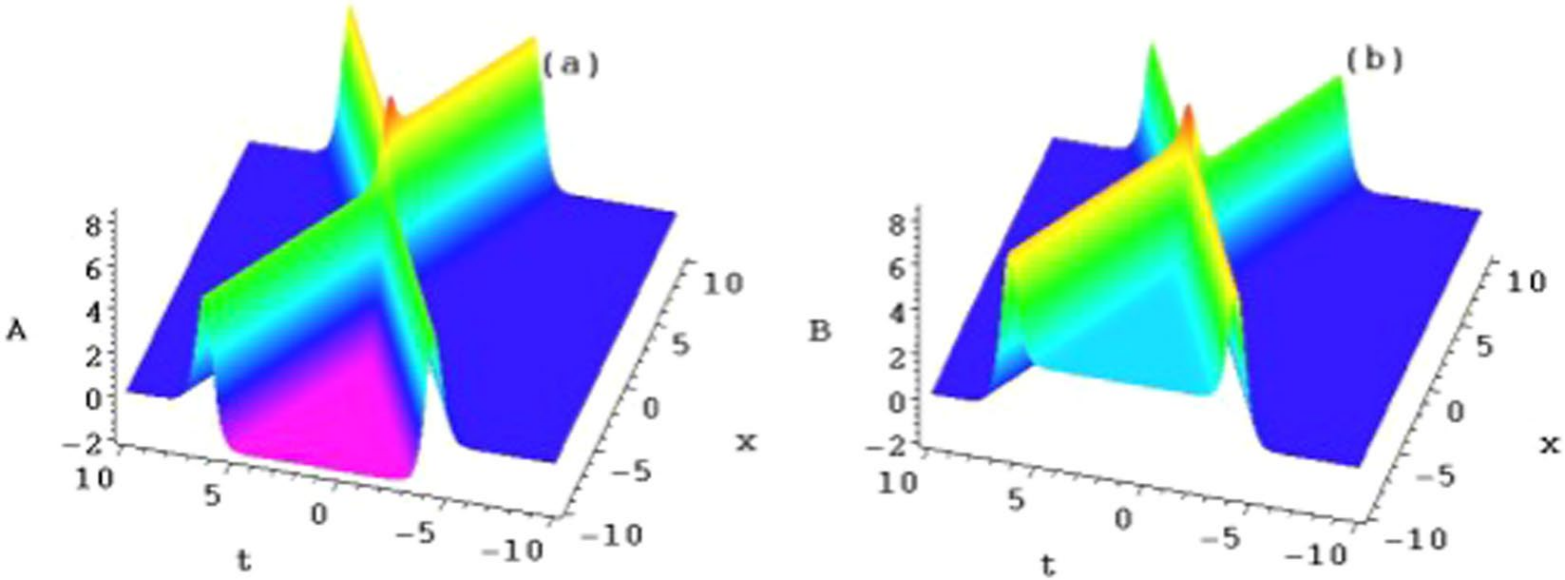
The interactions of the symmetry breaking line solitons of *A*_2_ and *B*_2_ of the solution (18).

**Figure 7:**
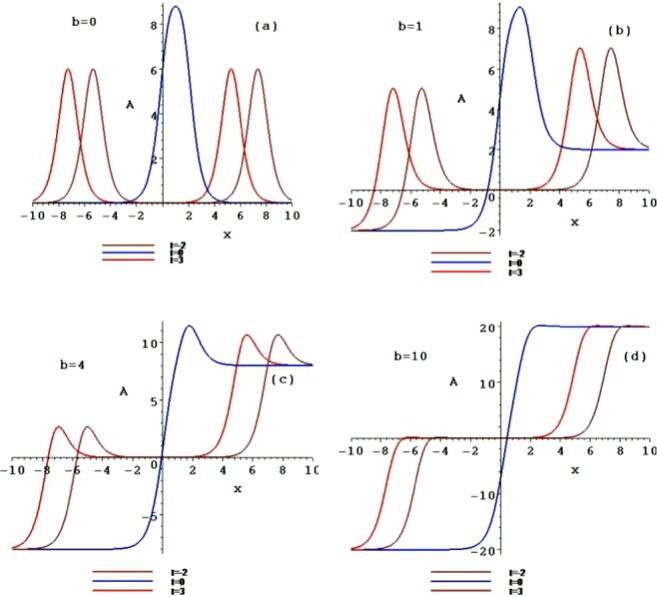
The contour plots of the interaction of the symmetry breaking line solitons *A*_2_ with $b=0,b=1,b=4$ and $b=10$ at different times $t=-\,2,1$ and 3, respectively.

**Figure 8:**
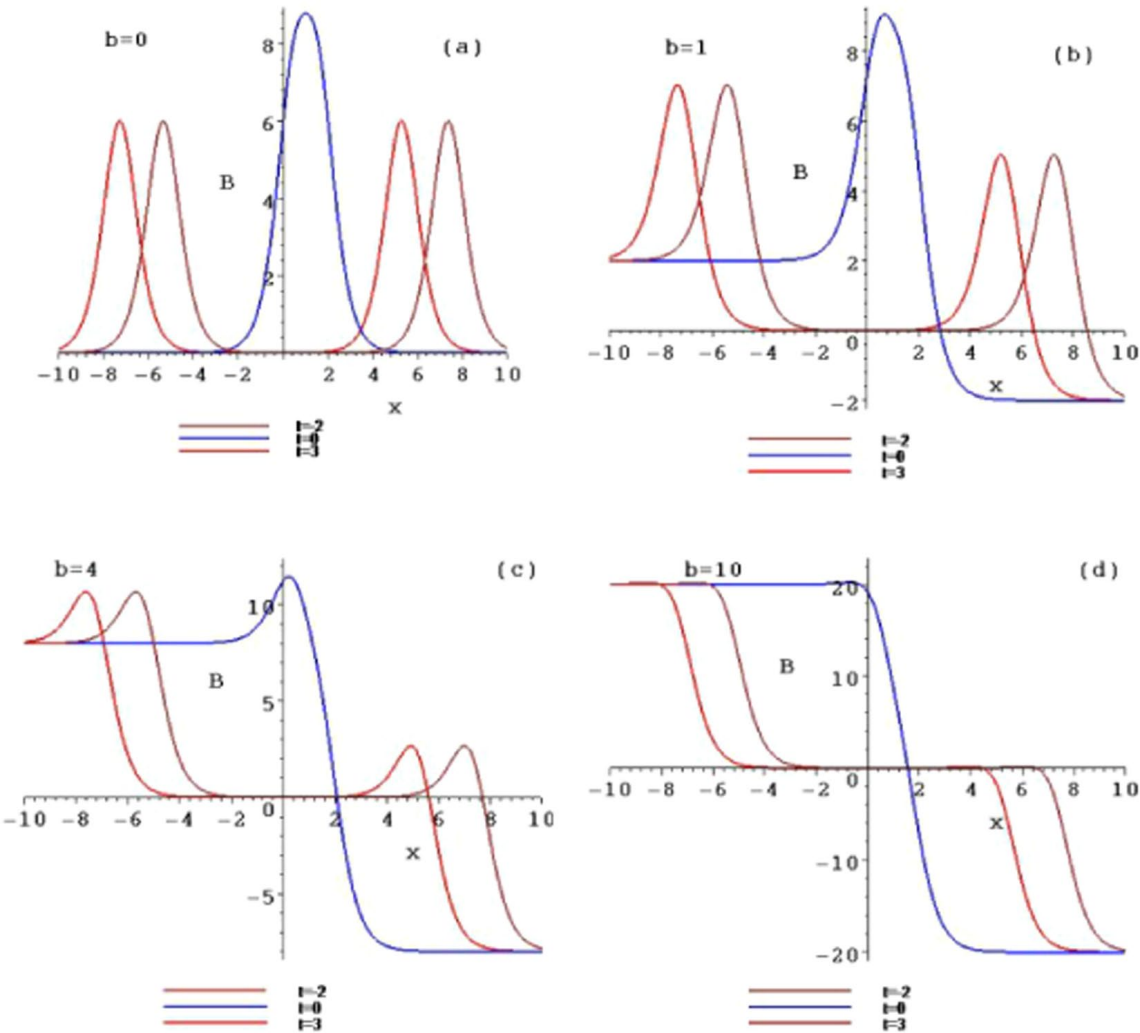
The contour plots of the interaction of the symmetry breaking line solitons *B*_2_ with $b=0,b=1,b=4$ and $b=10$ at different times $t=-\,2,1$ and 3, respectively.

This is the parity and time reversal invariant form of Eq. (16). For example, taking the constants $$b={k}_{1}={\delta }_{1}={\delta }_{2}=$$$${\delta }_{+}={\delta }_{-}=1,{k}_{2}=-\,1,{x}_{0}={t}_{0}=2,$$ two corresponding interactions of the symmetry breaking line solitons are presented (Fig. 6). Figures 7 and 8 are the contour plots of these structures in the $$(x,A/B)$$-plane, taking $$b=0,b=1,b=4$$ and $$b=10$$ at different times $$t=-\,2,1$$ and 3, respectively. As the parameter *b* and time *t* increases, the interactions of the symmetry breaking line soliton solutions of Eq. (4) also trend to two symmetry breaking kinks.

#### **Example 3**.**3**

Interaction of the triple symmetry breaking solitons

For $n=3$, the solution (14) possesses the following form

21 $$\begin{array}{lllll}f & \equiv  & {f}_{3} & = & {K}_{\{\}}\,\cosh ({\xi }_{1}+{\xi }_{2}+{\xi }_{3})\\  &  &  &  & +\,{K}_{\{1\}}\,\cosh ({\xi }_{1}-{\xi }_{2}-{\xi }_{3})\\  &  &  &  & +\,{K}_{\{2\}}\,\cosh ({\xi }_{1}-{\xi }_{2}+{\xi }_{3})\\  &  &  &  & +\,{K}_{\{3\}}\,\cosh ({\xi }_{1}+{\xi }_{2}-{\xi }_{3}),\end{array}$$

where

22a $${K}_{\{\}}={a}_{12}^{+}{a}_{13}^{+}{a}_{23}^{+},\,{K}_{\{1\}}={a}_{12}^{-}{a}_{13}^{-}{a}_{23}^{+},\,{K}_{\{2\}}={a}_{12}^{-}{a}_{13}^{+}{a}_{23}^{-},\,{K}_{\{3\}}={a}_{12}^{+}{a}_{13}^{-}{a}_{23}^{-},$$

22b $$\begin{array}{rcl}{a}_{ij}^{\pm } & = & \sqrt{2{k}_{i}^{2}+2{k}_{j}^{2}-{k}_{i}{k}_{j}({\delta }_{i}{\delta }_{j}\pm 3)},\\ {\xi }_{i} & = & {k}_{i}(x-\frac{{x}_{0}}{2})+2{\delta }_{i}{k}_{i}^{2}(t-\frac{{t}_{0}}{2}),\\ {\delta }_{i}^{2} & = & 1,\,(i=1,2,3).\end{array}$$

Therefore, the interaction of the triple symmetry breaking soliton solution of Eq. (4) can be expressed as

23 $${A}_{3}=\frac{6\gamma }{\beta }{(\mathrm{ln}{f}_{3})}_{xx}+b{(\mathrm{ln}{f}_{3})}_{x},\,{B}_{3}={A}_{3}(\,-\,x+{x}_{0},-\,t+{t}_{0}).$$

**Figure 9:**
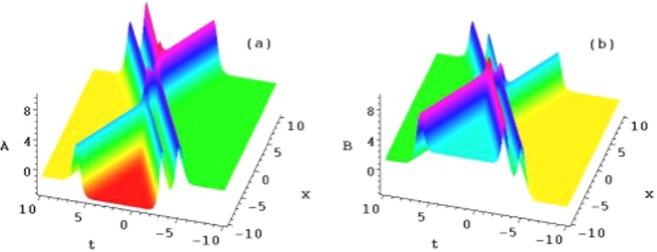
The interactions of the triple symmetry breaking solitons of *A*_3_ and *B*_3_ of the solution (23).

**Figure 10:**
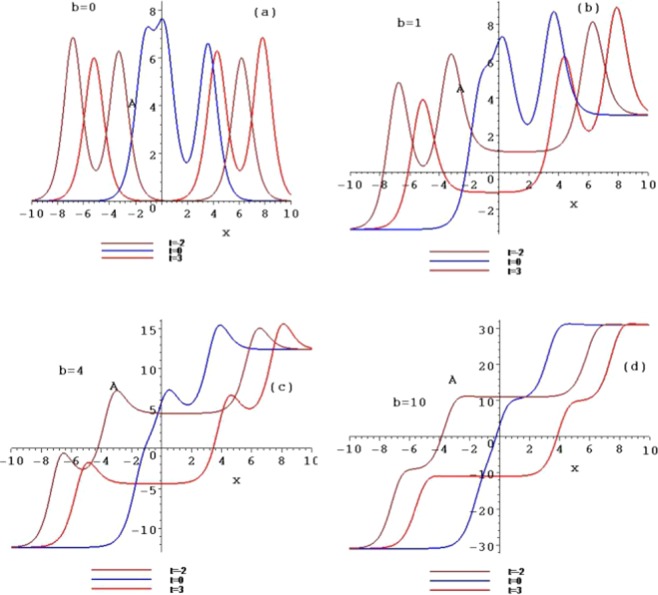
The contour plots of the interaction of the triple symmetry breaking solitons *A*_3_ with $b=0,b=1,b=4$ and $b=10$ at different times $t=-\,2,1$ and 3, respectively.

**Figure 11:**
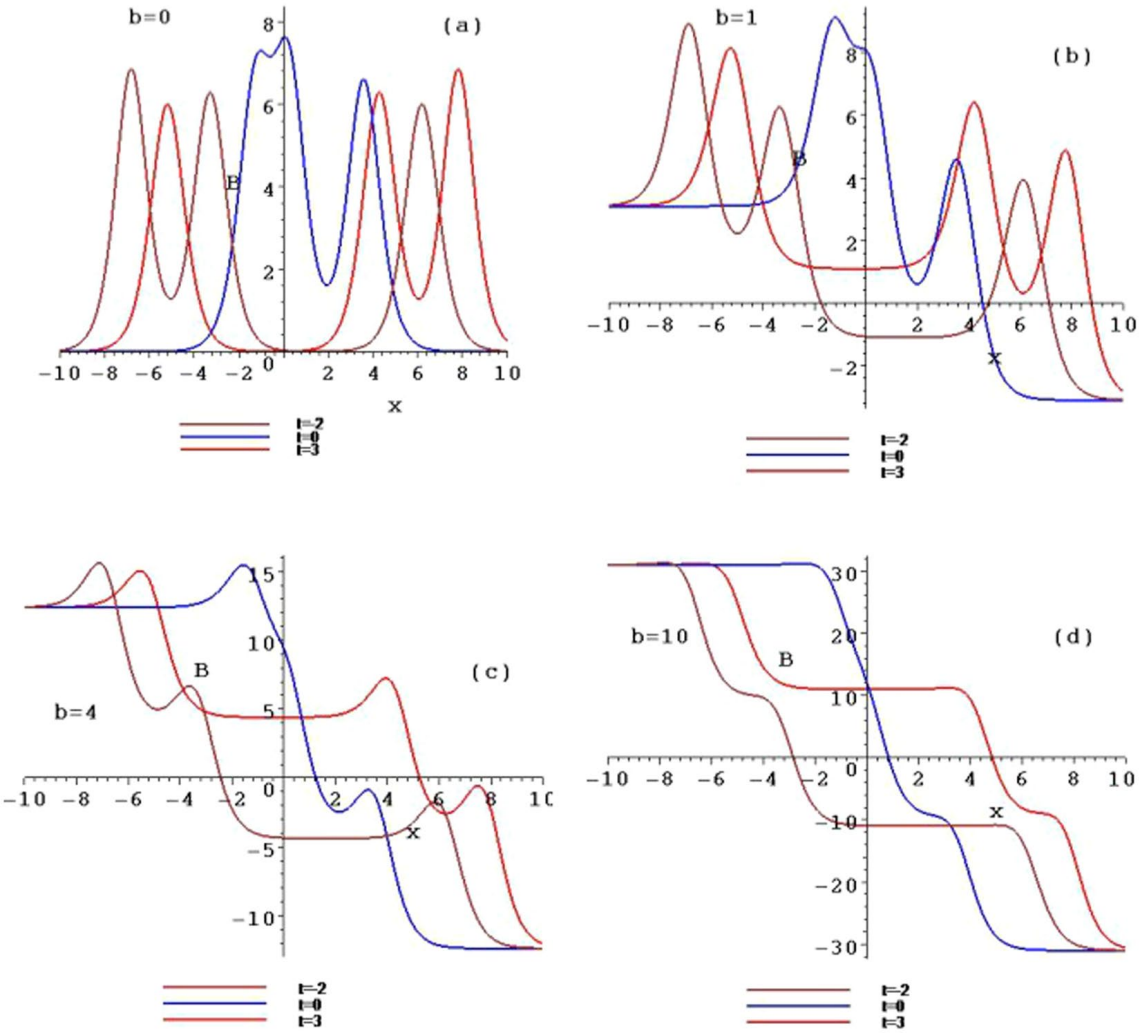
The contour plots of the interaction of the triple symmetry breaking solitons *B*_3_ with $b=0,b=1,b=4$ and $b=10$ at different times $t=-\,2,1$ and 3, respectively.

Figure 9 is two corresponding interactions of the triple symmetry breaking solitons when taking the constants $b={k}_{1}={\delta }_{1}={\delta }_{2}={\delta }_{3}=1,{k}_{2}=-\,1,{k}_{3}=-\,1.1,{x}_{0}={t}_{0}=2,$ and the parameters $\alpha =0,\beta =\gamma =-\,1$. Figures 10 and 11 are the contour plots of these structures in the $(x,A/B)$-plane, taking $b=0,b=1,b=4$ and $b=10$ at different times $t=-\,2,1$ and 3, respectively.

From these presented results of Examples 3.1–3.3, we have a different conclusion from the previous, that is “the multisoliton solutions with odd numbers and the multisoliton solutions with even numbers but with pursuant interactions are prohibited”^12^.

### Symmetry breaking breather solution

Based on the bilinear form (6) of the AB-Boussinesq system, the symmetry breaking breather solution of Eq. (4) can also be expressed as

24 $$A=\frac{6\gamma }{\beta }{(\mathrm{ln}f)}_{xx}+b{(\mathrm{ln}f)}_{x},\,B=A(\,-\,x+{x}_{0},-\,t+{t}_{0}),$$

when the bilinear function

25 $$\begin{array}{rcl}f & = & 2\sqrt{{b}_{1}}\,\cosh \{{b}_{3}[{a}_{1}(x-\frac{{x}_{0}}{2})+{a}_{2}(t-\frac{{t}_{0}}{2})]\}\\  &  & +\,{b}_{2}\,\cos \{{b}_{4}[{a}_{3}(x-\frac{{x}_{0}}{2})+{a}_{4}(t-\frac{{t}_{0}}{2})]\},\end{array}$$

and the coefficients

26 $$\begin{array}{rcl}{a}_{2} & = & -\,\frac{\sqrt{2}{a}_{1}{a}_{3}{b}_{4}(\alpha +2\gamma {R}_{2})}{{R}_{3}},\\ {a}_{4} & = & \frac{\sqrt{2}{R}_{3}}{2{b}_{4}},\\ {b}_{1} & = & \frac{{b}_{2}^{2}{R}_{3}^{2}[{R}_{3}^{2}-2{a}_{3}^{2}{b}_{4}^{2}(4{a}_{3}^{2}{b}_{4}^{2}\gamma -\alpha )]}{8{a}_{1}^{2}{b}_{3}^{2}[{R}_{3}^{2}(\alpha +4{a}_{1}^{2}{b}_{3}^{2}\gamma )+2{a}_{3}^{2}{b}_{4}^{2}{(\alpha +2\gamma {R}_{2})}^{2}]},\end{array}$$

and

27 $$\begin{array}{l}{R}_{1}={a}_{1}^{2}{b}_{3}^{2}+{a}_{3}^{2}{b}_{4}^{2},\\ {R}_{2}={a}_{1}^{2}{b}_{3}^{2}-{a}_{3}^{2}{b}_{4}^{2},\\ {R}_{3}=\sqrt{{R}_{2}\alpha +({R}_{2}^{2}-4{a}_{1}^{2}{a}_{3}^{2}{b}_{3}^{2}{b}_{4}^{2})\gamma +{R}_{1}\sqrt{({\alpha }^{2}+2{R}_{2}\alpha \gamma +{R}_{1}^{2}{\gamma }^{2})}}.\end{array}$$

## Residual symmetry of the AB-Boussinesq system

Recently, it was found that the residue in Painlevé truncated expansion corresponds to a nonlocal symmetry for the Painlevé integrable system and then such type of symmetry was referred to residual symmetry. This residual symmetry was localized to Lie point symmetry by introducing suitable prolonged system^30^.

From the leading term analysis of the AB-Boussinesq system (4), the Bäcklund transformation is extended as follows

28 $$A=\frac{6\gamma }{\beta }{(\mathrm{ln}f)}_{xx}+b{(\mathrm{ln}f)}_{x}+\tilde{A},\,B=\frac{6\gamma }{\beta }{(\mathrm{ln}f)}_{xx}-b{(\mathrm{ln}f)}_{x}+\tilde{B},$$

with

29 $$\begin{array}{l}\tilde{A}=\frac{3\gamma {f}_{xx}^{2}}{2\beta {f}_{x}^{2}}-\frac{2\gamma {f}_{xxx}}{\beta {f}_{x}}-\frac{{f}_{t}^{2}}{2\beta {f}_{x}^{2}}-\frac{\alpha }{2\beta }+p,\\ \tilde{B}=\frac{3\gamma {f}_{xx}^{2}}{2\beta {f}_{x}^{2}}-\frac{2\gamma {f}_{xxx}}{\beta {f}_{x}}-\frac{{f}_{t}^{2}}{2\beta {f}_{x}^{2}}-\frac{\alpha }{2\beta }-p,\end{array}$$

and $p\equiv p(x,t)$ is a constraint function to be determined later. The Schwarzian structure has two consistent conditions

30 $${C}_{t}+C{C}_{x}+\gamma {S}_{x}=0,$$

31 $${p}_{xx}=-\,\frac{b}{6\gamma }C{C}_{x}-\frac{b{f}_{xx}}{2{f}_{x}}S-\frac{b{f}_{xx}^{3}}{4{f}_{x}^{3}}-\frac{b}{3}{S}_{x},$$

where the variable quantities

32 $$C\equiv \frac{{f}_{t}}{{f}_{x}},\,S\equiv \frac{{f}_{xxx}}{{f}_{x}}-\frac{3{f}_{xx}^{2}}{2{f}_{x}^{2}}.$$

In other words, the coupled equations with the nonlocal symmetry functions *f* and *p* are

33 $${f}_{tt}=\frac{{f}_{xx}{f}_{t}^{2}}{{f}_{x}^{2}}+\frac{4\gamma {f}_{xx}{f}_{xxx}}{{f}_{x}}-\frac{3\gamma {f}_{xx}^{3}}{{f}_{x}^{2}}-\gamma {f}_{xxxx},$$

34 $${p}_{xx}=\frac{5b{f}_{xx}{f}_{xxx}}{6{f}_{x}^{2}}-\frac{b{f}_{xxxx}}{3{f}_{x}}-\frac{a{f}_{t}{f}_{xt}}{6\gamma {f}_{x}^{2}}+\frac{b{f}_{t}^{2}{f}_{xx}}{6\gamma {f}_{x}^{3}}-\frac{b{f}_{xx}^{3}}{2{f}_{x}^{3}}.$$

After introducing two auxiliary functions $g\equiv g(x,t)$ and $h\equiv h(x,t)$, which should obey the rules

35 $$g={f}_{x},\,h={g}_{x},$$

The enlarged AB-Boussinesq system of Eq. (4) according to Eqs. (4), (29), (33) and (34) given by

36a $${A}_{tt}+\alpha {A}_{xx}+\beta (A+B){A}_{xx}+\frac{1}{2}\beta {({A}_{x}+{B}_{x})}^{2}+\gamma {A}_{xxxx}=0,$$

36b $${B}_{tt}+\alpha {B}_{xx}+\beta (A+B){B}_{xx}+\frac{1}{2}\beta {({A}_{x}+{B}_{x})}^{2}+\gamma {B}_{xxxx}=0,$$

36c $$\alpha {f}_{x}^{2}-2\beta (p-A){f}_{x}^{2}+\gamma (4{f}_{x}{f}_{xxx}-3{f}_{xx}^{2})+{f}_{t}^{2}=0,$$

36d $$\alpha {f}_{x}^{2}+2\beta (p+B){f}_{x}^{2}+\gamma (4{f}_{x}{f}_{xxx}-3{f}_{xx}^{2})+{f}_{t}^{2}=0,$$

36e $${f}_{tt}{f}_{x}^{2}-{f}_{t}^{2}{f}_{xx}+\gamma (3{f}_{xx}^{3}+{f}_{x}^{2}{f}_{xxxx}-4{f}_{x}{f}_{xx}{f}_{xxx})=0,$$

36f $$b{f}_{t}({f}_{t}{f}_{xx}-{f}_{x}{f}_{xt})+\gamma (5b{f}_{x}{f}_{xx}{f}_{xxx}-2b{f}_{x}^{2}{f}_{xxxx}-6{f}_{x}^{3}{p}_{xx}-3b{f}_{xx}^{3})=0,$$

Under the transformations

37 $$\begin{array}{rcl}A & = & A+\epsilon {\sigma }^{A},\\ B & = & B+\epsilon {\sigma }^{B},\\ f & = & f+\epsilon {\sigma }^{f},\\ g & = & g+\epsilon {\sigma }^{g},\\ h & = & h+\epsilon {\sigma }^{h},\\ p & = & p+\epsilon {\sigma }^{p},\end{array}$$

with the infinitesimal parameter $\epsilon$, the linearized system of the enlarged AB-Boussinesq Eq. (36) reads

38a $${\sigma }_{tt}^{A}+\alpha {\sigma }_{xx}^{A}+\beta [({\sigma }_{x}^{A}+{\sigma }_{x}^{B})({A}_{x}+{B}_{x})+({\sigma }^{A}+{\sigma }^{B}){A}_{xx}+(A+B){\sigma }_{xx}^{A}]+\gamma {\sigma }_{xxxx}^{A}=0,$$

38b $${\sigma }_{tt}^{B}+\alpha {\sigma }_{xx}^{B}+\beta [({\sigma }_{x}^{A}+{\sigma }_{x}^{B})({A}_{x}+{B}_{x})+({\sigma }^{A}+{\sigma }^{B}){B}_{xx}+(A+B){\sigma }_{xx}^{B}]+\gamma {\sigma }_{xxxx}^{B}=0,$$

38c $${f}_{t}{\sigma }_{t}^{f}+\alpha {f}_{x}{\sigma }_{x}^{f}-\beta {f}_{x}({\sigma }^{p}{f}_{x}+2p{\sigma }_{x}^{f}-2A{\sigma }_{x}^{f}-{\sigma }^{A}{f}_{x})+\gamma (2{f}_{x}{\sigma }_{xxx}^{f}+2{f}_{xxx}{\sigma }_{x}^{f}-3{f}_{xx}{\sigma }_{xx}^{f})=0,$$

38d $${f}_{t}{\sigma }_{t}^{f}+\alpha {f}_{x}{\sigma }_{x}^{f}+\beta {f}_{x}({\sigma }^{p}{f}_{x}+2p{\sigma }_{x}^{f}+2B{\sigma }_{x}^{f}+{\sigma }^{B}{f}_{x})+\gamma (2{f}_{x}{\sigma }_{xxx}^{f}+2{f}_{xxx}{\sigma }_{x}^{f}-3{f}_{xx}{\sigma }_{xx}^{f})=0,$$

38e $$\begin{array}{c}{f}_{x}^{2}{\sigma }_{tt}^{f}+2{f}_{x}{f}_{tt}{\sigma }_{x}^{f}-{f}_{t}^{2}{\sigma }_{xx}^{f}-2{f}_{t}{f}_{xx}{\sigma }_{t}^{f}\\ \,+\,\gamma [(9{f}_{xx}^{2}-4{f}_{x}{f}_{xxx}){\sigma }_{xx}^{f}+(2{f}_{x}{f}_{xxxx}-4{f}_{xx}{f}_{xxx}){\sigma }_{x}^{f}\\ \,+\,{f}_{x}({f}_{x}{\sigma }_{xxxx}^{f}-4{f}_{xx}{\sigma }_{xxx}^{f})]=0,\end{array}$$

38f $$\begin{array}{c}\alpha [({f}_{x}{f}_{xt}-2{f}_{t}{f}_{xx}){\sigma }_{t}^{f}+{f}_{t}({f}_{x}{\sigma }_{xt}^{f}-{f}_{t}{\sigma }_{xx}^{f}+{f}_{xt}{\sigma }_{x}^{f})]\\ \,+\,\gamma [(6{f}_{x}^{2}{\sigma }_{xx}^{p}+2b{f}_{x}{\sigma }_{xxxx}^{f}-5b{f}_{xx}{\sigma }_{xxx}^{f}){f}_{x}\\ \,+\,(4b{f}_{x}{f}_{xxxx}-5b{f}_{xx}{f}_{xxx}+18{f}_{x}^{2}{p}_{xx}){\sigma }_{x}^{f}\\ \,+\,(9b{f}_{xx}^{2}-5b{f}_{x}{f}_{xxx}){\sigma }_{xx}^{f}]=0,\end{array}$$

38g $${\sigma }_{x}^{f}-{\sigma }^{g}=0,\,{\sigma }_{x}^{g}-{\sigma }^{h}=0.$$

Thus, the derived residual symmetry $\{{\sigma }^{A},{\sigma }^{B}\}$ has the local solution

39 $$\begin{array}{rcl}{\sigma }^{A} & = & bg+\frac{6\gamma h}{\beta },\\ {\sigma }^{B} & = & -bg+\frac{6\gamma h}{\beta },\\ {\sigma }^{f} & = & -{f}^{2},\\ {\sigma }^{g} & = & -2fg,\\ {\sigma }^{h} & = & -2(fh+{g}^{2}),\\ {\sigma }^{p} & = & bg.\end{array}$$

Meanwhile, by solving the following initial value problem

40a $$\begin{array}{rcl}\frac{\partial \hat{A}(\epsilon )}{\partial \epsilon } & = & \frac{6\gamma }{\beta }\hat{H}(\epsilon )+b\hat{G}(\epsilon ),\\ \frac{\partial \hat{B}(\epsilon )}{\partial \epsilon } & = & \frac{6\gamma }{\beta }\hat{H}(\epsilon )-b\hat{G}(\epsilon ),\\ \frac{\partial \hat{F}(\epsilon )}{\partial \epsilon } & = & -{\hat{F}}^{2}(\epsilon ),\end{array}$$

40b $$\begin{array}{rcl}\frac{\partial \hat{G}(\epsilon )}{\partial \epsilon } & = & -2\hat{F}(\epsilon )\hat{G}(\epsilon ),\\ \frac{\partial \hat{H}(\epsilon )}{\partial \epsilon } & = & -2[{\hat{G}}^{2}(\epsilon )+\hat{F}(\epsilon )\hat{H}(\epsilon )],\\ \frac{\partial \hat{P}(\epsilon )}{\partial \epsilon } & = & b\hat{G}(\epsilon ),\end{array}$$

40c $$\hat{A}(0)=A,\,\hat{B}(0)=B,\,\hat{F}(0)=f,\,\hat{G}(0)=g,\,\hat{H}(0)=h,\,\hat{P}(0)=p,$$

one can achieve the finite transformation theorem.

### Theorem

If $\{u,\,v,\,f,\,g,\,h,\,p,\,h\}$ is a solution of the extended system (4), (29), (33) and (34), so is $\{\hat{A}(\epsilon ),\,\hat{B}(\epsilon ),\,\hat{F}(\epsilon ),\,\hat{G}(\epsilon ),\,\hat{H}(\epsilon ),\,\hat{P}(\epsilon )\}$ with

41a $$\begin{array}{rcl}\hat{A}(\epsilon ) & = & A+\frac{b\epsilon g}{1+\epsilon f}+\frac{6\gamma \epsilon h}{\beta (1+\epsilon f)}-\frac{6\gamma {\epsilon }^{2}{g}^{2}}{\beta {(1+\epsilon f)}^{2}},\\ \hat{B}(\epsilon ) & = & B-\frac{b\epsilon g}{1+\epsilon f}+\frac{6\gamma \epsilon h}{\beta (1+\epsilon f)}-\frac{6\gamma {\epsilon }^{2}{g}^{2}}{\beta {(1+\epsilon f)}^{2}},\end{array}$$

41b $$\begin{array}{rcl}\hat{F}(\epsilon ) & = & \frac{f}{1+\epsilon f},\\ \hat{G}(\epsilon ) & = & \frac{g}{{(1+\epsilon f)}^{2}},\\ \hat{H}(\epsilon ) & = & \frac{h}{{(1+\epsilon f)}^{2}}-\frac{2\epsilon {g}^{2}}{{(1+\epsilon f)}^{3}},\\ \hat{P}(\epsilon ) & = & \frac{b\epsilon g}{1+\epsilon f}+p.\end{array}$$

where $\epsilon$ is an arbitrary group parameter.

## Summary and Conclusion

It is believed that there are some really novel phenomena in two-place nonlocal systems compared with local one. In this article, we studied the nonlocal Boussinesq equation coupled with AB systems. Furthermore, After introducing the bilinear variable transformation, the different expressions of function in the Bäcklund transformation are obtained. By choosing special parameters, the abundant solutions, including symmetry breaking rogue wave, soliton and breather solutions of the AB-Boussinesq system, are discussed in detail. At last, with the derived modified Bäcklund transformation, the residual symmetry of the nonlocal Boussinesq **system** is researched by introducing two auxiliary variables, and the finite symmetry transformation is obtained through solving the initial value problem. In brief, the variable coefficient with shifted parity and delayed time reversal in nonlocal AB- Boussinesq system is discussed, from which abundant of controllable symmetry breaking solutions are illustrated by changing parameters of events A and B. It is valuable to the nonlocal AB symmetric system analysis and the associated applications with mathematical models into more real physical systems for further study.
